# Cardiac arrest risk standardization using administrative data compared to registry data

**DOI:** 10.1371/journal.pone.0182864

**Published:** 2017-08-04

**Authors:** Anne V. Grossestreuer, David F. Gaieski, Michael W. Donnino, Joshua I. M. Nelson, Eric L. Mutter, Brendan G. Carr, Benjamin S. Abella, Douglas J. Wiebe

**Affiliations:** 1 Department of Emergency Medicine, Beth Israel Deaconess Medical Center, Boston, Massachusetts, United States of America; 2 Department of Emergency Medicine, Thomas Jefferson University, Philadelphia, Pennsylvania, United States of America; 3 Department of Medicine, Division of Pulmonary and Critical Care Medicine, Beth Israel Deaconess Medical Center, Boston, Massachusetts, United States of America; 4 Department of Emergency Medicine, University of Pennsylvania, Philadelphia, Pennsylvania, United States of America; 5 Department of Emergency Medicine, Queen’s University, Kingston, Ontario, Canada; 6 Center for Clinical Epidemiology and Biostatistics, University of Pennsylvania, Philadelphia, Pennsylvania, United States of America; Azienda Ospedaliero Universitaria Careggi, ITALY

## Abstract

**Background:**

Methods for comparing hospitals regarding cardiac arrest (CA) outcomes, vital for improving resuscitation performance, rely on data collected by cardiac arrest registries. However, most CA patients are treated at hospitals that do not participate in such registries. This study aimed to determine whether CA risk standardization modeling based on administrative data could perform as well as that based on registry data.

**Methods and results:**

Two risk standardization logistic regression models were developed using 2453 patients treated from 2000–2015 at three hospitals in an academic health system. Registry and administrative data were accessed for all patients. The outcome was death at hospital discharge. The registry model was considered the “gold standard” with which to compare the administrative model, using metrics including comparing areas under the curve, calibration curves, and Bland-Altman plots. The administrative risk standardization model had a c-statistic of 0.891 (95% CI: 0.876–0.905) compared to a registry c-statistic of 0.907 (95% CI: 0.895–0.919). When limited to only non-modifiable factors, the administrative model had a c-statistic of 0.818 (95% CI: 0.799–0.838) compared to a registry c-statistic of 0.810 (95% CI: 0.788–0.831). All models were well-calibrated. There was no significant difference between c-statistics of the models, providing evidence that valid risk standardization can be performed using administrative data.

**Conclusions:**

Risk standardization using administrative data performs comparably to standardization using registry data. This methodology represents a new tool that can enable opportunities to compare hospital performance in specific hospital systems or across the entire US in terms of survival after CA.

## Introduction

Cardiac arrest (CA) is a widespread and unexpected clinical condition that represents a challenge to prevent, manage, and study. Differences in definitions,[1] termination-of-resuscitation rules,[2, 3] data collection[1, 4] and participation in registries,[5] as well as patient heterogeneity[6] make even capturing the incidence of CA difficult.[1, 4, 7] This diversity can lead to differences in outcomes that may be influenced by variations in care. [8–21] Recent initiatives to change both intra- and post-arrest care have led to improved outcomes,[22–28] highlighting the importance of performing these assessments. Additionally, the National Academy of Medicine has recently recognized as a priority the need for better CA data collection and outcomes improvement.[29]

An important step to better understand the management of CA involves comparing hospitals.[30] Unfortunately, many US hospitals do not participate in a registry that provides such outcomes; contributing can be prohibitive in terms of financial and time costs.[31, 32] As of 2016, out of the over 5000 hospitals in the United States[33], only 7% were contributing to the largest in-hospital cardiac arrest registry in the country[34], and about 40% were providing outcomes to EMS agencies regarding out-of-hospital cardiac arrest patients treated by that EMS agency and delivered to their hospital.[35] Additionally, because these registries are voluntary, participation may lead to selection bias and other systematic errors.[36] As registry data are the only current method for risk adjustment[37] in CA, there is no way to enable fair comparison of observed mortality relative to expected mortality given patient characteristics across all US hospitals treating CA.

Quality is compared nationwide through the Centers for Medicare and Medicaid Services using administrative-type claims data to risk standardize the patient case mix of individual hospitals, which potentially could point to an avenue through which to measure quality in CA. To our knowledge, no studies have investigated whether administrative data on CA, shown to be useful for risk standardization of sepsis patients[38] and potentially available for all hospitals in the U.S., could perform as well as registry data to accomplish risk standardization to study variability in CA outcomes. If administrative-type data perform as well as registry data in this population, we will have evidence that a tool for risk standardization potentially can be developed and applied to hospitals to compare quality across the US. We therefore aimed to develop a method for risk-standardizing hospital survival after CA using administrative data that is validated against one using registry data.

## Methods

### Data source

The registry data were from the Penn Alliance for Therapeutic Hypothermia (PATH) database. PATH is an internet-based registry established by the University of Pennsylvania in 2010. PATH includes CA data from pre-hospital, emergency department, and in-hospital settings. Potentially available to any US hospital, PATH supports the tracking of all patients who experience CA and receive cardiopulmonary resuscitation. CA is defined in PATH as a loss of pulse with subsequent chest compressions. Each patient record in PATH consists of 30 required data elements based on the Utstein template.[4, 39] One hundred additional data elements are required for research participation. Further optional data elements are also available to address specific research questions. Data are entered via a secure website and maintained on a password-protected encrypted server at the University of Pennsylvania. Data are collected retrospectively at each of the participating institutions. Before entering data, data abstractors undergo structured training including mock case entry and case review. All participants are provided with a standardized data dictionary and are subject to a formal auditing process. PATH currently supports 34 member hospitals from 19 US states and includes data from over 5000 CAs. Exclusion criteria for this study were age <18 years, traumatic etiology of arrest, active do-not-resuscitate orders prior to arrest, and lack of administrative data.

Administrative data for this study were from the Penn Data Store, a research initiative at the University of Pennsylvania that integrates clinical data on all University of Pennsylvania Health System (UPHS) patients. All available administrative information on CA patients (defined as having an ICD-9 code of 427.5) seen at three UPHS hospitals, the Hospital of the University of Pennsylvania, Penn Presbyterian Medical Center, and Pennsylvania Hospital, was queried, and consisted of demographics, procedure codes, diagnosis codes, and drug and other orders. These data were then matched on medical record number to records from the PATH database. Only patients with both data in PATH and Penn Data Store were included in the risk standardization model building, creating a convenience sample of a granular dataset matched with administrative-type data. This study was approved by the University of Pennsylvania Institutional Review Board with a waiver of consent.

### Model building

Recommended guidelines for conducting risk adjustment for trauma, another time-sensitive critical illness, have been published, allowing comparison of trauma center outcomes.[40] Using these methods as guidance, we applied and adapted that approach to develop two risk standardization models.

First, we developed a method for risk-standardization using registry data for in- and out-of-hospital CA patients using logistic regression with survival to discharge as the outcome. Cox regression was not used because our interest was not in time to the outcome of interest (death) but in whether death had occurred by hospital discharge. A total of 12 variables (Table 1) were modeled as potential independent, adjustor variables. The variables were selected to match, to the extent possible, the non-modifiable variables in the Utstein template.[4, 39] These variables were modeled through a backward stepwise variable selection process (using a p<0.25 to enter the model)[41, 42] to generate the most parsimonious model and evaluate changes in predictive ability. Variables that did not contribute to prediction were excluded from the final model. The final model included race, whether the arrest was witnessed, initial rhythm, age, year of arrest, etiology of arrest, and whether the patient regained consciousness shortly post-arrest (defined as ineligibility for TTM due to purposeful following of commands). Significant missing data (more than 5% but no more than 15%) were addressed through multiple imputation, conducted using 20 iterations and then combined using the “mi estimate” Stata command.[40, 43, 44] A final logistic regression model generated the risk-adjusted predicted probability of death for each patient, ranging from 0 to 1, with higher values indicating a higher predicted probability that a given patient had died by hospital discharge. This final predictive model was assessed using conventional techniques including the Hosmer-Lemeshow goodness-of-fit statistic to assess calibration, calibration curves, c-statistic to assess discrimination, and Akaike information criterion value to compare model fit and composition across multiple models. The resulting model was considered the gold standard for risk adjustment for our study purposes. To accommodate multiple datasets created through multiple imputation, we used two strategies, after arriving at a final model, to derive a predicted probability for each patient: the predicted value of each imputed dataset averaged per patient and the predicted value of the imputed dataset closest to the median area under the curve with the better Hosmer-Lemeshow goodness-of-fit statistic.

**Table 1:** Variables explored for registry risk standardization.

Age*	Sex*	Race*
Location of arrest*	Etiology of arrest	Initial pulseless rhythm*
If patient was transferred*	Year of arrest*	If arrest was witnessed*
Duration of arrest	Bystander CPR provided*	Whether patient regained consciousness shortly post-arrest*

*variables with a univariate p-value with the outcome of interest of <0.25

Next, we developed a method for risk-standardizing hospital survival using administrative data. To identify candidate variables for exploration, we queried all available diagnostic codes, procedure codes, demographics, and orders for all patients with an ICD-9 code for cardiac arrest (427.5). We then isolated all unique diagnosis codes, procedure codes, and orders. These were assessed by two physician-fellows in resuscitation science to determine, by consensus, which of these should be explored as candidate variables due to their possible relationship to survival. Only the non-modifiable factors, as assessed by the physician-fellows, associated with CA were included. This was to limit controlling for features that are modifiable such as treatment with targeted temperature management could adjust away differences in care that are due to aspects of hospital performance that we would like to identify. Each identified candidate variable then was tested in univariate logistic regression against the outcome of interest (death at hospital discharge).

We next employed the same logistic regression methodology to the administrative data as was employed with the registry data, developing a logistic regression model using death at hospital discharge as the outcome. The administrative candidate variables were modeled as potential independent, adjustor variables through a manual forward stepwise variable selection process (using a univariate p<0.25 to enter the model).[41, 42] Variables that did not contribute to prediction were excluded from the final model. Variance inflation factors were checked and collinear variables were collapsed or omitted. There was no missing data in the administrative data set that was greater than 1%. The final logistic regression model generated the risk-adjusted predicted probability of death for each patient; this model was used in comparison to the “gold standard” registry model.

Finally, we assessed the performance of the risk standardization done using administrative data to the performance of the “gold standard” risk standardization done using registry data. The results for both sets of analysis were reported as c-statistics, calibration plots, and Bland-Altman plots. To evaluate the models against each other, we used Bland-Altman plots to assess mean difference in predicted values and the percentage of values outside the limits of agreement, defined as two standard deviations of the mean difference,[45] a Hosmer-Lemeshow plot of the performance of the predicted values from each model compared to the observed values, and tests of the equality of receiver operating characteristic (ROC) areas between models. The last assessment, a test of the equality of ROC areas between models, was chosen a priori as the final determination for model comparison, with significance assessed at p<0.05.

## Results

### Patient population

There were 2453 patients who had both administrative and registry data between 1/2000-4/2015 with 1820 (74.2%) having the outcome of interest. This cohort had a median age of 63 (IQR: 51, 74) years; 57.8% of these patients were male, 44.1% white, 24.8% had an initial shockable rhythm of ventricular fibrillation or pulseless ventricular tachycardia (VF/VT), 60.6% had a presumed cardiac etiology of arrest, 53.7% had an out-of-hospital CA (OHCA), 26.3% of OHCAs received bystander CPR, 74.8% had a witnessed arrest, and 83.5% had intra-arrest epinephrine given, with a median dose of 2 (IQR: 0, 3) mg. The median duration of arrest was 11 (IQR: 5, 25) minutes, 62.5% had return of spontaneous circulation (ROSC), 19.8% of patients received TTM, 17.4% of patients regained arousal shortly post-ROSC, and 20.0% of patients had a favorable neurologic outcome (as defined as a Cerebral Performance Category [CPC] score of 1–2).[39, 46–48]

### Registry risk standardization

There were 2622 cardiac arrests in PATH between 1/2000-4/2015 and 2453 of the arrests matched with administrative data (93.6%). The patients that did not match with administrative data were significantly more likely to have initial shockable rhythms, to be African-American, to have a cardiac etiology of arrest, to have an OHCA, to have a witnessed arrest, to not receive epinephrine intra-arrest and to receive a lower dose if given, to have a longer duration of arrest, to achieve ROSC, to not receive TTM, to regain consciousness shortly post-arrest, to survive to hospital discharge and to have an CPC score of 1 or 2 at hospital discharge (S1 Table).

The final registry risk standardization model, when limited to non-modifiable variables, contained six predictor variables (whether the arrest was witnessed, initial rhythm, age, year of arrest, and whether the patient regained consciousness post-arrest). Evaluating the risk standardization models using Bland-Altman plots, we found a much worse fit in terms of Pitman’s test of difference in variance when using the model composed of the average predicted values from all imputations. Therefore, we chose to use the values from the median imputed dataset as the “gold standard.” The c-statistic in the final model was 0.8097 (95% CI: 0.7882–0.8311) with a Hosmer-Lemeshow goodness-of-fit statistic of 0.51.

### Administrative risk standardization

Penn Data Store included 5424 patients between 1/2000-4/2015 with an ICD-9 code of 427.5. These patients were 57.3% male, 50.1% white, and had a median length of hospital stay of 6 (IQR: 1, 17) days. Forty-five percent of the patients with an ICD-9 code for CA were matched with registry data. On these 5424 arrests, there were 1423 unique procedure codes, 2001 unique drugs, 5632 unique orders, and 4723 unique diagnosis codes (13,792 candidate variables including race, sex, and age).

A list of all unique procedure codes, drug orders, other orders, and diagnosis codes was compiled for assessment by two physician-fellows in resuscitation science involved in this study. Both fellows eliminated any variables assessed as irrelevant for predicting survival outcome in CA patients. Any variable eliminated by one fellow but not the other remained eligible for exploration. After the fellows’ assessment, 1719 (12.5%) potential variables remained. Each of these was then analyzed in univariate logistic regression with survival to discharge as the outcome. Any variable with a p-value of <0.25 remained eligible for the model, which resulted in 317 variables. Using manual forward selection in order of lowest p-value to highest, variables then entered the logistic regression model. Variables remained in the model if they improved the predictive value and were removed if they worsened the predictive value or if it remained the same. Of the 317 candidate variables with a p<0.25 in univariate association with death, 170 were identified as being non-modifiable. After analyzing these variables, 97 remained in the model (S2 Table) with a c-statistic of 0.8182 (95% CI: 0.7987–0.8377) and a Hosmer-Lemeshow goodness of fit of 0.11. Due to concerns of overfitting, we did a post hoc assessment of the minimal number of variables that would be required for a c-statistic of 0.8, which was 48 (S3 Table).

### Comparing risk standardization models

**Fig 1:**
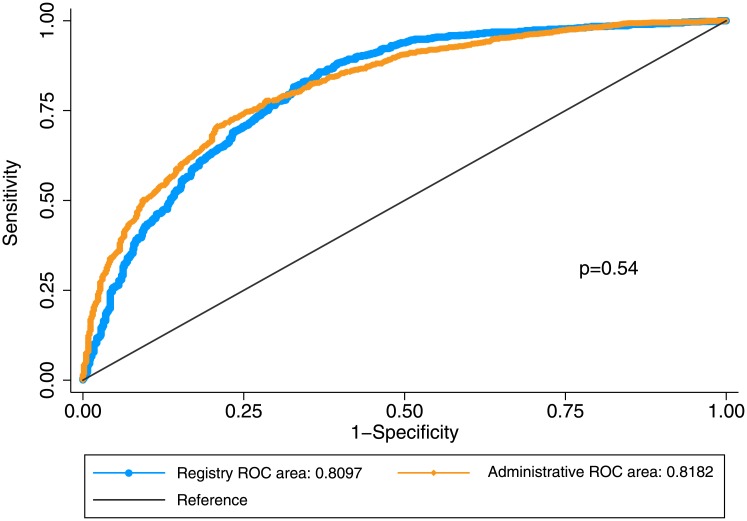
Comparison of ROC curves between registry and administrative data.

**Fig 2:**
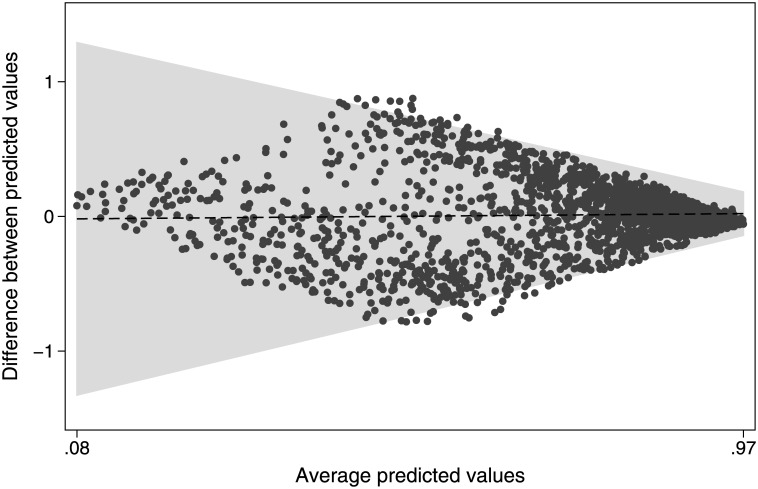
Bland Altman Plot of agreement between registry and administrative risk standardization models.

**Fig 3:**
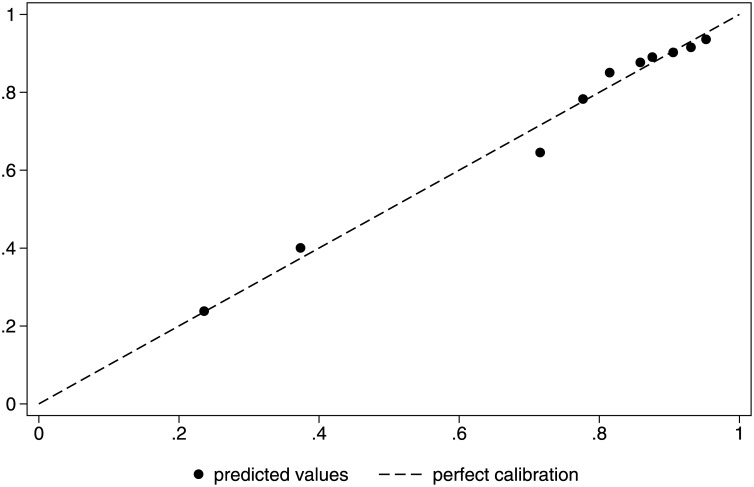
Calibration plot for registry data in all patients.

**Fig 4:**
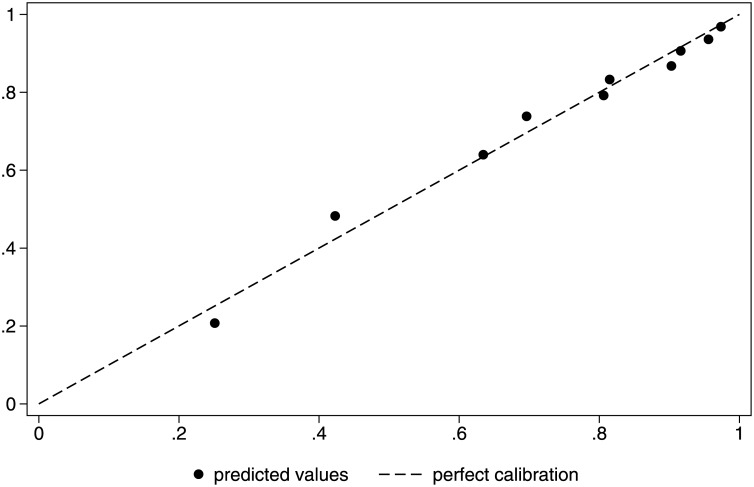
Calibration plot for administrative data in all patients.

Using the “rocgold” Stata command to compare the two models, there was an insignificant difference (p = 0.542; Fig 1). Controlling for trend with a Bland-Altman plot, we found that the mean difference between the two methods of risk standardization was -0.02 (95% CI: -0.06–0.02) with a non-significant Pitman's test of difference in variance (p = 0.13), which we conclude represents good agreement; the line of equality falls within the confidence interval of the mean difference and only 1.81% of the data points lie outside the recommended range of two standard deviations of the mean difference (Fig 2). Both models had good calibration, as seen in Figs 3 and 4 and by non-significant Hosmer-Lemeshow goodness-of-fit statistics.

## Discussion

In developing two risk standardization models with extremely small differences between their c-statistics (0.0085), we have identified that risk adjustment modeling for CA can be performed using administrative data, which are readily available and less costly[31, 32] and less challenging to compile and to access than registry data. We therefore have evidence that a tool developed using administrative data is feasible and has the potential to be used for quality assessment in cardiac arrest. This tool also could be applied in research to identify variability in the management of CA and to learn from effective modalities and protocols to allow hospitals identify opportunities for improvement.

In the U.S., there is an estimated 42% difference in the odds of survival in in-hospital arrests by hospital even after risk adjusting the patient population for comparison.[49] Hospital-level interventions have been shown to be effective,[22–28] and hospitals that perform well with regard to in-hospital CA have also been found to be better at preventing CA.[50] Therefore, adequate comparisons, such as those provided using risk standardization, are vital to improve patient care and outcomes.

A recent study called into question the utility of administrative data for identifying out-of-hospital CA.[51] Investigators queried ICD-9 codes for cardiac arrest as well as VF, paroxysmal ventricular tachycardia, ventricular flutter, and respiratory arrest and found that only 40% of patients who had these ICD-9 codes had an out-of-hospital CA upon chart review. However, 94% of the CAs in our registry matched to administrative data, although there were some significant differences between the patients who were matched and those who were not. Despite our ability to risk standardize in a comparable way to registry data in our convenience sample of cardiac arrest patients, further work is needed to develop methods to identify this population in administrative datasets as well as to generalize to a larger sample from more than one health system.

The data from PATH have the limitations of data from any retrospective registry, including the use of predefined data points and the risk of data entry errors or inconsistencies. Additionally, while administrative data potentially are available from all institutions and can reflect “real world” situations, the information in these databases are not collected for research purposes, and often key variables are not recorded by administration, which have non-medical and non-research motivations for collecting information; these motivations can lead to documentation that might not match with research documentation. Finally, the data collected by the University of Pennsylvania Health System may differ from that collected at other institutions, despite having many common elements, limiting generalizability.

This study serves as evidence that risk standardization using administrative data is comparable to that of registry data in the context of CA. The critical gap of only having information on the performance of a subset of hospitals that participate in a registry potentially could be addressed by providing support for a new method that may identify hospital performance and variability. Next steps include identifying a set of administrative variables that consistently provide discriminatory and well-calibrated risk standardization from a set that are most likely to be completely collected at hospitals and easily extractable for analysts performing research and quality comparisons. Future investigations into expanding this methodology to include more sites may lead to a new tool for nationwide risk standardization to allow benchmarking and comparison of hospitals in terms of expected to observed mortality to identify high- and low-performing hospitals.

## Supporting information

S1 Table Patient demographics in registry data. (DOCX)

S2 Table Administrative data elements used in risk standardization model. (DOCX)

S3 Table Administrative data elements used in risk standardization model with C-Statistic of 0.8. (DOCX)
