# Supplementary material for: Cardiac arrest risk standardization using administrative data compared to registry data
Source: PLoS One. 2017 Aug 4;12(8):e0182864. doi: 10.1371/journal.pone.0182864 (PMC5544239; doi:10.1371/journal.pone.0182864)
Supplement: S1 Table — (DOCX) [file pone.0182864.s001.docx]

|  | Matched  N=2453 | Not Matched  N=169 | p-value |
| --- | --- | --- | --- |
| Age (median) | 63 (51, 74) | 63 (51, 74) | 0.668 |
| Male | 1409 (57.8) | 97 (57.4) | 0.915 |
| Race |  |  |  |
| White | 967 (44.1) | 52 (31.1) |  |
| Black | 1088 (49.6) | 99 (59.3) | 0.003 |
| Other | 140 (6.4) | 16 (9.6) |  |
| Initial Rhythm |  |  |  |
| Asystole | 604 (26.2) | 40 (26.5) |  |
| PEA | 1129 (49.0) | 59 (39.1) | 0.017 |
| VF/VT | 571 (24.8) | 52 (34.4) |  |
| Cardiac Etiology of Arrest | 899 (60.6) | 124 (75.6) | <0.001 |
| OHCA | 1305 (53.7) | 125 (74.4) | <0.001 |
| Witnessed Arrest | 1066 (74.8) | 136 (82.9) | 0.022 |
| Bystander CPR (OHCA only) | 144 (26.3) | 28 (26.9) | 0.899 |
| Epinephrine Given | 1031 (83.5) | 118 (73.3) | 0.001 |
| Epinephrine Dose (median) | 2 (0, 3) | 1 (0, 3) | 0.049 |
| Duration of Arrest (median)* | 11 (5, 26) | 21 (10, 34) | <0.001 |
| ROSC achieved | 1532 (62.5) | 169 (100.0) | <0.001 |
| TTM Performed* | 473 (19.8) | 133 (11.5) | <0.001 |
| Patient Regained Consciousness after ROSC* | 356 (17.4) | 35 (31.3) | <0.001 |
| Survival to Hospital Discharge | 633 (25.8) | 73 (43.2) | <0.001 |
| CPC at Hospital Discharge | 486 (20.0) | 65 (38.5) | <0.001 |

*only calculated on patients with ROSC
