# Supplementary material for: Cardiac arrest risk standardization using administrative data compared to registry data
Source: PLoS One. 2017 Aug 4;12(8):e0182864. doi: 10.1371/journal.pone.0182864 (PMC5544239; doi:10.1371/journal.pone.0182864)
Supplement: S2 Table — (DOCX) [file pone.0182864.s002.docx]

| Year of arrest | Age | Acute necrosis of liver (570) |
| --- | --- | --- |
| Obstructive sleep apnea (327.23) | History of tobacco use (V15.82) | Food/vomit pneumonitis (507) |
| Coma (780.01) | Bacteremia (790.7) | Sepsis (995.91) |
| Human immunodeficiency virus [HIV] disease (042) | Pure hypercholesterolemia (272) | Dissection of thoracic aorta (441.01) |
| Persistent vegetative state (780.03) | Chronic kidney disease (585.9) | Accidental poisoning –psychstimulant (E85.42) |
| Rheumatic heart failure (398.91) | Malignant neoplasm of ovary and other uterine adnexa (183) | Toxic encephalopathy (349.82) |
| Anaphylactic shock (995) | E. coli infection (041.4) | [Lymphoid leukemia](http://www.icd9data.com/2012/Volume1/140-239/200-209/204/204.htm) (204) |
| Hypoxemia (799.02) | Race | Pseudomonas (482.1) |
| Compression of brain (348.4) | Cerebral edema (348.5) | Urinary tract infection (599) |
| Secondary malignant neoplasm of respiratory and digestive systems (197) | Methicillin-resistant Staphylococus aureus septicemia (038.11) | Acute venous embolism and thrombosis of other specified veins (453.8) |
| Candidiasis of lung (112.4) | Atrioventricular block (426.1) | Sepsis (order) |
| Acute or chronic combined systolic and diastolic heart failure (428.43) | Acute myocardial infarction of inferoposterior wall, initial episode of care (410.31) | Mobitz (type) II atrioventricular block (426.12) |
| Cardiogenic shock (785.51) | Hemopericardium (423) | Long QT syndrome (426.82) |
| Aortic valve disorders (424.1) | Aortic atherosclerosis (440) | Fluid overload (276.69) |
| Critical illness myopathy (359.81) | Respiratory system disease (519.8) | Cerebral embolism with cerebral infarction (434.11) |
| Other specified disorders of circulatory system (459.89) | Pneumonia due to escherichia coli [E. coli] (482.82) | Congestive heart failure (428) |
| Coronary atherosclerosis of native coronary artery (414.01) | Chronic ischemic heart disease (414.8) | Occlusion and stenosis of carotid artery (433.1) |
| Interruption of the vena cava (38.7) | Delirium due to conditions classified elsewhere (293) | Ventricular fibrillation (427.41) |
| Other specified alveolar and parietoalveolar pneumonopathies (516.8) | Chronic venous embolism and thrombosis of internal jugular veins (453.76) | History of major cardiovascular surgery (V15.1) |
| Sinoatrial node dysfunction (427.81) | Chronic systolic heart failure (428.22) | Acute on chronic systolic heart failure (428.23) |
| Subarachnoid hemorrhage (430) | Hemiplegia/hemiparesis (438.2, 342.9) | Influenza with pneumonia (487) |
| Closed fracture of rib(s) (807) | Acute kidney failure (584.9) | Pulmonary collapse (518) |
| Chronic stomach ulcer with hemorrhage (531.4) | Dependence on respirator, status (V46.11) | Alcohol detoxification (94.62) |
| Syncope and collapse (780.2) | Retention of urine (788.2) | Streptococcal septicemia (38) |
| Leukocytosis (288.6) | Cocaine abuse (305.6) | Encephalopathy (348.3) |
| Primary pulmonary hypertension (416) | Pulmonary valve disorder (424.3) | Complete atrioventricular block (426) |
| Proteus infection (41.6) | Cirrhosis of liver (571.5) |  |
| Acute diastolic heart failure (428.31) | History of pulmonary embolism (V12.55) | Adult body mass index between 45.0-49.9 (V85.42) |
| Paroxysmal ventricular tachycardia (427.1) | Dissection of aorta, thoracoabdominal (441.03) | Abdominal aneurysm without mention of rupture (441.4) |
| Acute venous embolism and thrombosis of upper extremity (453.83) | Acute venous embolism and thrombosis of other thoracic veins (453.87) | Takotsubo syndrome (429.83) |
| Systemic inflammatory response syndrome due to noninfectious process without acute organ dysfunction (995.93) | Staphylococcus infection in conditions classified elsewhere and of unspecified site, other staphylococcus (041.19) | Friedländer's bacillus infection in conditions classified elsewhere and of unspecified site (041.3) |
| Other specified bacterial infections in conditions classified elsewhere and of unspecified site, other gram-negative organisms (041.85) | Personal history of (corrected) congenital malformations of heart and circulatory system (V13.65) | Pseudomonas infection in conditions classified elsewhere and of unspecified site (041.7) |
| Any poisoning (963.0, 963.1, 964.2, 965.00, 965.02, 965.09, 965.8, 967.0, 967.8, 969.03, 969.3, 969.7, 969.72, 970.81, 971.2, 971.3, 972.6, E85.04, E85.1, E85.29, E85.42, E85.55, E85.56, E85.82, E85.89, E868.9, E95.00, E95.01, E95.04, E980.2) | | |
