# Supplementary material for: Cardiac arrest risk standardization using administrative data compared to registry data
Source: PLoS One. 2017 Aug 4;12(8):e0182864. doi: 10.1371/journal.pone.0182864 (PMC5544239; doi:10.1371/journal.pone.0182864)
Supplement: S3 Table — (DOCX) [file pone.0182864.s003.docx]

| Year of arrest | Age | Bacteremia (790.7) |
| --- | --- | --- |
| Obstructive sleep apnea (327.23) | Pure hypercholesterolemia (272) | Food/vomit pneumonitis (507) |
| Hypoxemia (799.02) | Race | Sepsis (995.91) |
| Persistent vegetative state (780.03) | Chronic kidney disease (585.9) | Dissection of thoracic aorta (441.01) |
| Rheumatic heart failure (398.91) | Chronic ischemic heart disease (414.8) | Toxic encephalopathy (349.82) |
| Anaphylactic shock (995) | Aortic atherosclerosis (440) | Fluid overload (276.69) |
| Compression of brain (348.4) | Cardiogenic shock (785.51) | Urinary tract infection (599) |
| Acute venous embolism and thrombosis of other specified veins (453.8) | Chronic venous embolism and thrombosis of internal jugular veins (453.76) | Closed fracture of rib(s), unspecified (807.00) |
| Takotsubo syndrome (429.83) | Long QT syndrome (426.82) | Sepsis (order) |
| Acute or chronic combined systolic and diastolic heart failure (428.43) | Coronary atherosclerosis of native coronary artery (414.01) | Mobitz (type) II atrioventricular block (426.12) |
| Interruption of the vena cava (38.7) | Delirium due to conditions classified elsewhere (293) | Ventricular fibrillation (427.41) |
| Sinoatrial node dysfunction (427.81) | Chronic systolic heart failure (428.22) | Acute on chronic systolic heart failure (428.23) |
| Acute kidney failure (584.9) | Pulmonary collapse (518) | Hemiplegia (342.90) |
| Chronic stomach ulcer with hemorrhage (531.4) | Dependence on respirator, status (V46.11) | Complete atrioventricular block (426) |
| Syncope and collapse (780.2) | Retention of urine (788.2) | Encephalopathy (348.3) |
| Systemic inflammatory response syndrome due to noninfectious process without acute organ dysfunction (995.93) | Staphylococcus infection in conditions classified elsewhere and of unspecified site, other staphylococcus (041.19) | Pseudomonas infection in conditions classified elsewhere and of unspecified site (041.7) |
